# Does body size predict the buzz‐pollination frequencies used by bees?

**DOI:** 10.1002/ece3.5092

**Published:** 2019-03-21

**Authors:** Paul A. De Luca, Stephen Buchmann, Candace Galen, Andrew C. Mason, Mario Vallejo‐Marín

**Affiliations:** ^1^ School of Chemistry, Environmental & Life Sciences University of the Bahamas Nassau Bahamas; ^2^ Department of Ecology & Evolutionary Biology University of Arizona Tucson Arizona; ^3^ Department of Entomology University of Arizona Tucson Arizona; ^4^ Division of Biological Sciences University of Missouri Columbia Missouri; ^5^ Department of Biological Sciences University of Toronto Scarborough Toronto Ontario Canada; ^6^ Biological & Environmental Sciences University of Stirling Stirling UK

**Keywords:** Anthophila, buzz ratio, floral vibrations, frequency, *Pedicularis*, *Solanum*

## Abstract

Body size is an important trait linking pollinators and plants. Morphological matching between pollinators and plants is thought to reinforce pollinator fidelity, as the correct fit ensures that both parties benefit from the interaction. We investigated the influence of body size in a specialized pollination system (buzz‐pollination) where bees vibrate flowers to release pollen concealed within poricidal stamens. Specifically, we explored how body size influences the frequency of buzz‐pollination vibrations. Body size is expected to affect frequency as a result of the physical constraints it places on the indirect flight muscles that control the production of floral vibrations. Larger insects beat their wings less rapidly than smaller‐bodied insects when flying, but whether similar scaling relationships exist with floral vibrations has not been widely explored. This is important because the amount of pollen ejected is determined by the frequency of the vibration and the displacement of a bee's thorax. We conducted a field study in three ecogeographic regions (alpine, desert, grassland) and recorded flight and floral vibrations from freely foraging bees from 27 species across four families. We found that floral vibration frequencies were significantly higher than flight frequencies, but never exceeded 400 Hz. Also, only flight frequencies were negatively correlated with body size. As a bee's size increased, its buzz ratio (floral frequency/flight frequency) increased such that only the largest bees were capable of generating floral vibration frequencies that exceeded double that of their flight vibrations. These results indicate size affects the capacity of bees to raise floral vibration frequencies substantially above flight frequencies. This may put smaller bees at a competitive disadvantage because even at the maximum floral vibration frequency of 400 Hz, their inability to achieve comparable thoracic displacements as larger bees would result in generating vibrations with lower amplitudes, and thus less total pollen ejected for the same foraging effort.

## 1 INTRODUCTION

Body size is an important ecological trait that influences many aspects of an individual's relationship with other organisms and to its environment (Chown & Gaston, 2010; White, Ernest, Kerkhoff, & Enquist, 2007; Woodward et al., 2005). Body size is also important in mutualistic networks such as pollination systems, where a pollinator's body size may influence flower—pollinator matching, pollen transfer efficiency, and pollinator behavior. For instance, morphological matching between pollinators and flowers is thought to help reinforce pollinator fidelity to a host because the correct fit ensures that both parties benefit maximally from the interaction, that is resource extraction for the pollinator and successful pollen transfer for the plant (Anderson, Pauw, Cole, & Barrett, 2016; Anderson, Terblanche, & Ellis, 2010; Harder, 1985; Solis‐Montero & Vallejo‐Marin, 2017). In bees, body size can also influence specific behaviors related to foraging activity, including foraging distance (Greenleaf, Williams, Winfree, & Kremen, 2007; Zurbuchen et al., 2010) and efficiency (i.e., amount of pollen or nectar collected per unit time) (Peat, Tucker, & Goulson, 2005). The effect of body size can thus affect plant‐pollinator interactions at a variety of levels, from functional interactions based on morphology to patterns of pollen flow and pollinator behavior. Investigations into the association of body size and plant–pollinator interactions are particularly timely, as recent studies have demonstrated shifts in pollinator body size (or in a functional trait correlated with body size) are currently occurring in many habitats resulting from climate change (Miller‐Struttmann et al., 2015) and landscape simplification (Renauld, Hutchinson, Loeb, Poveda, & Connelly, 2016).

Buzz‐pollination (also referred to as “floral sonication”) is an excellent system for investigating body size effects on the behavioral interactions between pollinators and their host plants. Here, pollinators (mainly bees) extract pollen by mechanically vibrating the stamens where pollen is kept concealed inside modified (poricidal) anthers or corollas (Buchmann, 1983; Macior, 1968; Vallejo‐Marín, 2019). Buzz‐pollination is performed by female bees (Anthophila) in thousands of species, having evolved at least 45 times within the group (Cardinal, Buchmann, & Russell, 2018). Furthermore, about 6% of flowering plants comprising approximately 22,000 species are thought to be buzz‐pollinated (Buchmann, 1983; De Luca & Vallejo‐Marín, 2013). Typically, a female bee will bite the base of the anthers, curl the ventral side of her body against them and rapidly contract her thoracic indirect flight muscles (Harder & Barclay, 1994; King, 1993; Macior, 1968). Contraction of the flight muscles results in vertical (up‐down) displacement of the thoracic sternites and tergites, with the resulting vibrations being transmitted through the head, legs and body of the bee and into the poricidal structures, where the pollen grains inside are then expelled through pores in the tips (Buchmann & Hurley, 1978; Harder & Barclay, 1994; King & Lengoc, 1993).

A key property of floral sonication vibrations is fundamental frequency, which refers to the lowest frequency in the vibration. Its value (which usually ranges between 100 and 400 Hertz, Hz) results from the contraction rate of the thoracic indirect flight muscles and the tension the muscles apply to the exoskeleton (King, 1993; King & Buchmann, 2003; King, Buchmann, & Spangler, 1996). Body size is expected to greatly influence floral vibration frequency as a result of the constraints it places on the indirect flight muscles that control the production of these vibrations. In insects, an inverse relationship between body size and flight frequency exists such that larger insects beat their wings less rapidly, and thus use lower frequencies, than smaller‐bodied insects when flying (Ellington, 1985; Josephson, Malamud, & Stokes, 2000; Molloy, Kyrtatas, Sparrow, & White, 1987; Pringle, 1949). This imposes a size‐specific lower limit on flight frequency in order to keep an individual aloft (Byrne, Buchman, & Spangler, 1988; Casey, May, & Morgan, 1985), but whether floral vibration frequencies are similarly constrained by body size has not been widely explored. This is important because frequency is thought to play a key role affecting pollen release through its effect on how efficiently stamens vibrate (King & Buchmann, 1995,1996; King & Lengoc, 1993).

Some studies suggest that stamens release more pollen at frequencies above those produced by buzz‐pollinating bees, which is thought to limit the amount of pollen that can be extracted (Arceo‐Gómez, Martinez, Parra‐Tabla, & Garcıa‐Franco, 2011; Harder & Barclay, 1994). Others argue that no relationship exists between vibration frequency and the amount of pollen extracted (De Luca et al., 2013; King & Buchmann, 1996; Rosi‐Denadai, Araújo, Oliveira Campos, Cosme, & Guedes, 2018). Furthermore, a recent study proposed that because smaller bees, by virtue of having a smaller thorax, are unable to achieve as great a displacement of their thorax when vibrating flowers as larger bees, for a given frequency value they are unable to generate floral vibrations with amplitudes (power) comparable to bigger bees (Corbet & Huang, 2014). The amplitude, *A*, (quantified in acceleration, m/s^2^) of a floral vibration is determined by the equation: *A *= 2 × (pi^2^) × (*F* ^2^) × *D*, where *F* is the fundamental frequency (in Hz) and *D* is the displacement (in mm) (Buchmann & Hurley, 1978; King & Buchmann, 1996). Corbet and Huang (2014) argued that smaller bees might compensate for having a low thoracic displacement (*D*) by instead increasing the frequency (*F*) of a sonication vibration, thereby achieving an acceleration (*A*) equivalent to larger bees. Amplitude is positively correlated with pollen release (De Luca et al., 2013; Harder & Barclay, 1994; Rosi‐Denadai et al., 2018), therefore bees might be expected to maximize the amplitude of their floral vibrations in order to collect as much pollen as possible for their foraging effort. This raises the interesting prediction that when visiting the same floral resource, smaller bees should use higher frequencies than larger bees to produce floral vibrations with comparable amplitudes, and thus achieve the same level of high pollen ejection.

The few studies that have investigated the relationship between body size and floral vibration frequency offer mixed results. Studies within a single species of bumblebee (*Bombus* spp.) foraging on a single host plant reveal no significant relationship (De Luca et al., 2013; De Luca, Cox, & Vallejo‐Marín, 2014; Nunes‐Silva, Hrncir, Shipp, Kevan, & Imperatriz‐Fonseca, 2013), while two other studies that examined a single species of *Bombus* spp. on multiple plant species found that floral vibration frequency was either positively or negative correlated with body size depending on the metric that was used (i.e., mass or intertegular distance) (Corbet & Huang, 2014; Switzer & Combes, 2017). Most recently, Arroyo‐Correa, Beattie, and Vallejo‐Marín (2019) found that floral vibration frequency was positively associated with bee size in two species of *Bombus* spp. foraging on two types of *Solanum* flowers. Two studies, however, that expanded the focus to include several bee species across different families and genera foraging on the same host plant provide more compelling results. Burkart, Lunau, and Schlindwein (2011) measured flight and floral vibrations in 15 bee species from eastern Brazil visiting two species of *Solanum* flowers. They found that bee size was significantly negatively correlated with flight frequency as expected, but the slope of the relationship with floral vibration frequency was much flatter and non‐significant, indicating that floral vibrations across species do not scale with body size as flight frequencies do. In the other study, Rosi‐Denadai et al. (2018), also working in Brazil, measured floral vibration frequencies in 12 bee species foraging on a single species of *Solanum*. Although these researchers did not evaluate the relationship between body size and flight frequency, they did find that body size was also not significantly correlated with floral vibration frequency across species. Whether the findings from Brazil represent a general pattern across bees within the buzz‐pollination syndrome (Dellinger et al., 2018), or a result specific to bees in a tropical habitat is unknown, as data from other environments and pollinator assemblages is currently lacking. Accordingly, expanding the focus to include a diverse assortment of bees from multiple habitats is necessary in order to determine the generality of the relationship between body size and floral vibration frequency, and thus clarify its role in shaping pollen collection behavior within this pollination syndrome.

We investigated whether the lack of an association between body size and floral vibration frequency observed in Brazil (Burkart et al., 2011; Rosi‐Denadai et al., 2018) also holds for other assemblages of bees and plants in different ecogeographic regions. We measured body size–vibration frequency relationships from a wide array of buzz‐pollinating bees in three distinct environments that differed in their composition of both bee and plant taxa: An alpine community in the Rocky Mountains, Colorado, USA, an arid desert zone community in Arizona and New Mexico, USA, and a grassland habitat in southern Ontario, Canada. Our study addressed the following questions: (a) What is the range of bee species and body sizes visiting buzz‐pollinated plant species in different habitats (*Pedicularis parryi* and *P. groenlandica*; alpine community, *Solanum elaeagnifolium*; desert community, *S. dulcamara*; grassland community)? (b) What are the frequency characteristics of both flight and floral vibrations obtained from acoustic field recordings of foraging bees? (c) Is there a negative relationship between frequency and body size for flight and floral vibrations? (d) Do smaller bees use higher vibration frequencies than larger bees when visiting the same floral resource? By exploring body size effects on floral vibrations in bees spanning a wide range of body sizes and taxonomic identities across multiple environments, we have compiled a large dataset that broadens our knowledge of the role body size plays in influencing buzz‐pollination behavior, and in doing so we offer a framework for encouraging future research.

## 2 MATERIALS AND METHODS

### 2.1 Study sites

**Figure 1:**
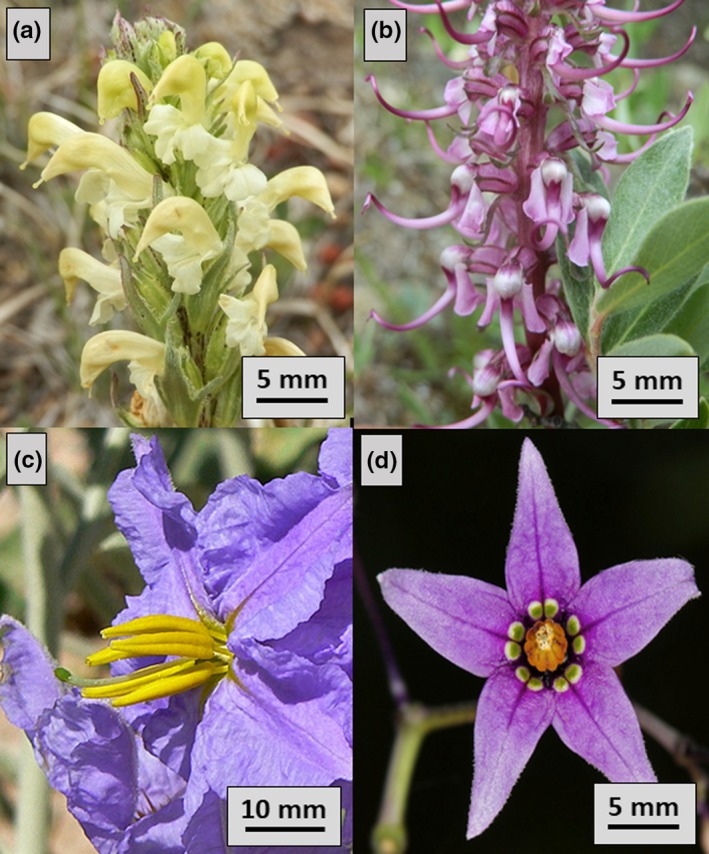
Buzz‐pollinated flowers sampled in this study. (a) *Pedicularis parryi*. (b) *P. groenlandica*. (c) *Solanum elaeagnifolium*. (d) *S. dulcamara*. Note the scale bar in each photo

We sampled for bees in three types of habitats: (a) a high elevation alpine site in Colorado, (b) three desert sites in south‐eastern Arizona extending into New Mexico, and (c) a grassland‐prairie site in southern Ontario, Canada. In Colorado, we made visual searches of foraging bees from July 1–14, 2017 in an open field under Pennsylvania Mountain (Park Co., CO, USA; N 39.25374°, W 106.11460°; elevation: 3,570 m). At this location there were two buzz‐pollinated plant species within the Orobanchaceae that bees visited: *Pedicularis parryi* (Parry's lousewort; Figure 1a) and *P. groenlandica* (Elephant's head lousewort; Figure 1b). In Arizona and New Mexico, we made visual searches from July 19 to 30, 2017 at three locations: (a) the grounds of the Southwestern Research Station (Cochise Co., AZ, USA; N 31.88330°, W 109.20547°; elevation: 1,596 m), (b) an open field at Cave Creek Ranch (Cochise, Co., AZ, USA; N 31.90488°, W 109.15582°; elevation: 1,427 m), and (c) along the roadside on Highway 338 south of Highway 9 in Animas, NM (Hidalgo Co., NM, USA; N 31.93292°, W 108.80575°, 1,337 m). At these locations, we observed bees visiting only one buzz‐pollinated plant species within the Solanaceae: *Solanum elaeagnifolium* (Silver‐leaf nightshade; Figure 1c). In southern Ontario, we made visual searches from July 5 to 20, 2018 at the Koffler Scientific Reserve (King City, Ontario, Canada; N 44.02990°, W 79.53337°, elevation: 305 m). At this location we observed bees visiting only one buzz‐pollinated plant within the Solanaceae: *S. dulcamara* (Bitter‐sweet nightshade; Figure 1d).

### 2.2 Recording floral sonication and flight vibrations

The production of floral sonication vibrations results in an audible sound as a by‐product of the vibrations radiating off the exoskeleton of the bee and into the surrounding air (Buchmann, 1983; Macior, 1968). Accordingly, a microphone can be used to record these sounds which can then be analyzed for their spectral and temporal parameters. A recent study confirmed that acoustic measures of the duration and fundamental frequency of floral sonication vibrations serve as reliable proxies for the true vibrational values of these parameters (see: De Luca, Giebink, Mason, Papaj, & Buchmann, 2018). We made visual searches of foraging bees beginning after sunrise and ending by the mid‐afternoon as bee activity declined. When we observed a bee approaching a flower, we followed it and held a digital acoustic recorder (either a Tascam DR‐100 MK‐III [TEAC America, Inc., Montebello, CA, USA] or Zoom H4 [Zoom North America, Hauppauge, NY, USA]), within 1–5 cm of the bee when it landed on the flower, always directing the microphone head to the dorsal surface of its thorax. We adjusted the microphone gain as needed to compensate for environmental sources of noise (e.g., wind, machinery, passing vehicles, and animals) in order to maximize the signal to noise ratio of recordings without causing over‐distortion. Recordings were saved as wave files (24‐bit, 48 kHz sampling rate). Bees were not disturbed by our presence and readily vibrated flowers. For most bees, we were also able to record flight vibrations as a bee either approached or departed a flower. In these cases, we held the microphone as close to the dorsal surface of the bee's thorax as possible (usually within 5 cm) for several seconds without disturbing it or interrupting its flight path. We then netted the bee and stored it in a chilled vial. The bee was later euthanized by freezing and then pinned for identification and to make intertegular distance (ITD) measurements (see below).

### 2.3 Analyzing floral sonication and flight vibrations

**Figure 2:**
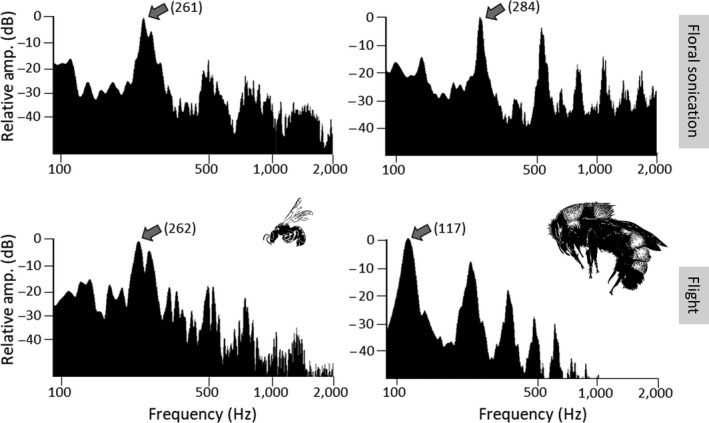
Example flight and sonication frequencies of bees sampled in this study. Top panels show floral sonication spectra and bottom panels show flight spectra. Left panels are of a small halictid bee (*Dialictus deludens*) and right panels a large apid bee (*Bombus sonorus*). Gray arrows point to the fundamental frequency with the exact value given in parentheses. Illustrations depicting each bee are scaled in size relative to each other. Note log scale used on the *x*‐axis

We used Audacity v. 2.1.3 (https://sourceforge.net/projects/audacity/) to measure the fundamental frequency (in Hertz, Hz) of floral sonication and flight vibrations, and the duration (in seconds) of floral sonication vibrations. We define fundamental frequency as the lowest frequency in a vibration (flight or floral) with the largest peak amplitude value when visualized in a Fast Fourier Transform (FFT) spectrum (see Figure 2). We define duration as the length of a single floral vibration. We first high‐pass filtered (100 Hz cut‐off, 12 dB per octave roll‐off) recordings to minimize the presence of low frequency noise and then we used the “Plot Spectrum” function (FFT = 8,192 Hz, Hamming window) to measure the peak frequency value. Our high‐pass cut‐off value of 100 Hz was low enough that it did not remove any relevant frequencies in flight or sonication vibrations. In some cases, it was difficult to distinguish frequencies of floral vibrations from sources of noise exhibiting similar frequency ranges when viewed in the FFT spectrum. In these instances, we verified that we were correctly measuring relevant frequency components by examining a spectrogram of the recording using the “Spectrogram” function (FFT = 8,192 Hz, Hamming window) in Audacity. Since spectrograms plot frequency as a function of time, it was possible to distinguish between distinct sources of sound and thus identify frequencies of floral sonication vibrations from non‐relevant sounds (e.g., wind, machinery, passing vehicles and animals).

### 2.4 Body size measurements

For each captured bee we measured its intertegular distance (ITD) as an indicator of body size (Cane, 1987). For bees captured in 2017, we used a Zeiss Stemi SV6 dissecting microscope (Carl Zeiss, Inc., Thornwood, NY, USA) set at 1.0× magnification that was fitted with an AxioCam 105 digital camera (Carl Zeiss, Inc., Thornwood, NY, USA) to take a digital photograph of the dorsal surface of the bee's thorax. The field of view of the microscope was calibrated so that we could measure the ITD from the digital photograph using Zen2 (“blue edition”) imaging software (Carl Zeiss Microscopy GmbH, 2011). For bees captured in 2018, we used a Performance Tools (model 80152) digital Vernier caliper to measure a bee's ITD while viewing the specimen under a Leica MZ16A dissecting microscope (Leica Microsystems, Inc., Buffalo Grove, IL, USA) set at 10× magnification.

### 2.5 Statistical analyses

To evaluate relationships between vibration properties and bee size, we first excluded data from unidentified bees without ITD measurements (i.e., recorded bees that escaped capture) and bees for which we did not also obtain flight vibration recordings. Furthermore, because of the limited sample sizes for bees within the Colletidae (one species, one individual) and Andrenidae (two species, two individuals) we excluded these families from further analysis. We conducted separate analyses for each of the three plant taxa (*Solanum elaeagnifolium*, *Pedicularis* spp., and *S. dulcamara*), which corresponded to the three ecogeographic regions (New Mexico/Arizona, Colorado and Ontario, respectively). We decided against analyzing all plants together as there was no overlap in bee species from different ecogeographic regions, and thus it was not possible to statistically compare floral sonication vibrations from the same bee species foraging on plants from different environments. We also combined the data for both *Pedicularis* species, as preliminary analysis revealed no significant differences in floral vibration properties between the two congeners.

We used a linear mixed‐effect model to analyze the relationship between the frequency of flight and floral sonication vibrations and bee size. We used individual vibrations (from flight and floral sonication) as our experimental unit. Bee family, vibration type (floral or flight), bee size (ITD) and the interaction vibration type*bee size were included as fixed effects. We included family as a fixed effect because we were interested in determining the effect of family in the characteristics of flight and floral buzzes. Bee genus, bee species and bee individual were included as nested random effects, which allowed us to account for the non‐independence of individuals from the same species and genus and of multiple vibrations of both types produced by the same bee. The relationship between floral vibration duration and bee size was analyzed using a linear mixed‐effect model with ITD and bee family as fixed effects, and genus, species and individual as nested random effects.

In order to examine whether smaller bees raised floral vibration frequency more than larger bees, we calculated a “buzz ratio” for each bee, defined as the frequency value during floral vibration divided by the flight frequency value (Burkart et al., 2011; Corbet & Huang, 2014). Recall that the production of floral vibrations occurs by contraction of the thoracic flight muscles. Therefore, comparing floral vibration frequencies between bees needs to account for the fact that individual bees have a unique flight frequency value (determined by their size) which represents the baseline from which its floral vibration frequency is derived (Gilmour & Ellington, 1993; King & Buchmann, 2003). Accordingly, buzz ratios offer a standardized way to evaluate size‐related differences between bees in their ability to generate floral vibration frequencies of a particular value. A single buzz ratio was calculated for each individual bee (our experimental unit) based on its average floral vibration and flight frequency values. The relationship between buzz ratio and bee size was then analyzed using a linear mixed‐effect model with bee size and family as fixed effects, and bee species and genus as nested random effects. All models were analyzed in R v. 3.5.1 (R Core Team, 2018) using the packages lme4 (Bates, Maechler, & Bolker, 2014), lmerTest (Kuznetsova, Brockhoff, & Christensen, 2016) and sjPlot (Lüdeke, 2017). For all analyses, we used a normal error distribution (lmer function in lme4) and verified that the residuals of the models had an approximately normal distribution.

## 3 RESULTS

We recorded a total of 877 floral sonication vibrations from 187 bees of which we captured 121. We identified 27 species representing four families (Apidae—14 species, Halictidae—10 species, Andrenidae—2 species, Colletidae—1 species; Table 1). Our subsequent analyses, however, focused on bees within the Apidae and Halictidae only, and only included bees for which we had an ITD measurement and recordings of both flight and floral sonication vibrations. Accordingly, our reduced dataset included 659 floral sonication vibrations and 352 flight vibrations. For *S. elaeagnifolium*, we analyzed 400 individual buzzes (302 floral and 98 flight) from 34 bees from 13 species in seven genera in the families Apidae and Halictidae. For *Pedicularis* spp., we analyzed 315 individual buzzes (154 floral and 161 flight) from 44 bees from five species of *Bombus* (Apidae). For *S. dulcamara*, we analyzed 296 individual buzzes (203 floral and 93 flight) from 37 bees from five species in four genera in the families Apidae and Halictidae. The average number of sonication vibrations per bee was 5 (range: 1–20) on *S. elaeagnifolium*, 3 (range: 1–9) on *Pedicularis* spp. and 4 (range: 1–16) on *S. dulcamara*.

**Table 1:** Bee species sampled in this study

Species	Family	Location	*N*	Floral frequency (Hz)	Floral duration (s)	ITD (mm)
*Agapostemon femoratus *(Agasp)	Halictidae	Arizona/New Mexico	4 (42)	264 ± 15	0.58 ± 0.15	2.76 ± 0.03
*Anthophora terminalis* (Anterm)	Apidae	Southern Ontario	2 (8)	307 ± 3	1.3 ± 0.58	3.9 ± 0.13
*Augochlora pura* (Augpur)	Halictidae	Southern Ontario	6 (36)	292 ± 16	0.45 ± 0.14	2.02 ± 0.19
*Augochloropsis metallica* (Augmet)	Halictidae	Southern Ontario	4 (30)	230 ± 23	1.52 ± 0.81	2.67 ± 0.03
*Bombus bifarius* (Bbif)	Apidae	Colorado	1 (1)	269	0.54	5.75
*B. flavifrons* (Bflv)	Apidae	Colorado	1 (5)	321 ± 9	0.27 ± 0.09	5.53
*B. impatiens* (Bimpat)	Apidae	Southern Ontario	24 (125)	289 ± 30	1.03 ± 0.57	4.68 ± 0.22
*B. melanopygus* (Bmel)	Apidae	Colorado	22 (74)	348 ± 25	0.82 ± 0.25	4.25 ± 0.41
*B. mixtus* (Bmix)	Apidae	Colorado	8 (37)	327 ± 25	0.66 ± 0.17	4.35 ± 0.72
*B. sylvicola* (Bsyl)	Apidae	Colorado	12 (37)	341 ± 19	1.25 ± 0.77	3.95 ± 0.18
*B. morrisoni* (Bmor)	Apidae	Arizona/New Mexico	2 (15)	301 ± 19	0.72 ± 0.01	6.05 ± 0.76
*B. sonorous* (Bson)	Apidae	Arizona/New Mexico	21 (114)	287 ± 17	0.79 ± 0.22	5.42 ± 0.45
*B. vagans* (Bvagan)	Apidae	Southern Ontario	1 (4)	285 ± 12	1.37 ± 1.08	3.96
*Dialictus deludens* (Diasp)	Halictidae	Arizona/New Mexico	1 (16)	257 ± 41	0.64 ± 0.22	1.04
*Dialictus pseudotegulare *(Diasp)	Halictidae	Arizona/New Mexico	1 (11)	287 ± 16	0.27 ± 0.14	1.07
*Dialictus *“new species” (Diasp)	Halictidae	Arizona/New Mexico	1 (6)	308 ± 13	0.55 ± 0.09	1.23
*Dialictus* sp. (Diasp)	Halictidae	Arizona/New Mexico	1 (7)	259 ± 11	0.45 ± 0.16	1.24
*Exomalopsis solani* (Exsol)	Apidae	Arizona/New Mexico	1 (5)	191 ± 13	0.89 ± 0.39	2.62
*Lassioglossum *sp.*	Halictidae	Arizona/New Mexico	1 (10)	169 ± 13	1.00 ± 0.45	2.6
*Melissodes *sp. (Melsp)	Apidae	Arizona/New Mexico	5 (40)	281 ± 32	0.78 ± 0.46	3.2 ± 0.09
*Nomia foxii* (Nfox)	Halictidae	Arizona/New Mexico	5 (24)	254 ± 21	0.58 ± 0.11	2.81 ± 0.12
*N. tetrazonata* (Ntet)	Halictidae	Arizona/New Mexico	1 (3)	258 ± 17	0.70 ± 0.31	2.86
Protandrena mexicanorum*	Andrenidae	Arizona/New Mexico	1 (3)	285 ± 83	0.76 ± 0.55	2.18
Ptiloglossa sp.*	Colletidae	Arizona/New Mexico	1 (20)	290 ± 22	1.31 ± 0.55	5.75
Protoxaea gloriosa*	Andrenidae	Arizona/New Mexico	1 (4)	329 ± 17	0.66 ± 0.17	5.97
*Xylocopa c. arizonensis* (Xca)	Apidae	Arizona/New Mexico	2 (4)	256 ± 25	1.77 ± 1.50	7.76 ± 0.24
*X. varipuncta* (Xvar)	Apidae	Arizona/New Mexico	4 (15)	251 ± 7	0.80 ± 0.25	8.31 ± 0.16

Note

Abbreviations for species appearing in Figures 4, 5, 6, 7 are given in parentheses after the species name. Descriptive statistics for floral vibration properties and intertegular distance (ITD) are provided as the mean ± *SD*. *N* = total number of individuals sampled with the value in parentheses denoting the number of floral sonication vibrations that were obtained for that species. Asterisk denotes taxa that were excluded from the statistical analysis. See text for details.

### 3.1 Frequency of floral and flight vibrations and bee size

**Figure 3:**
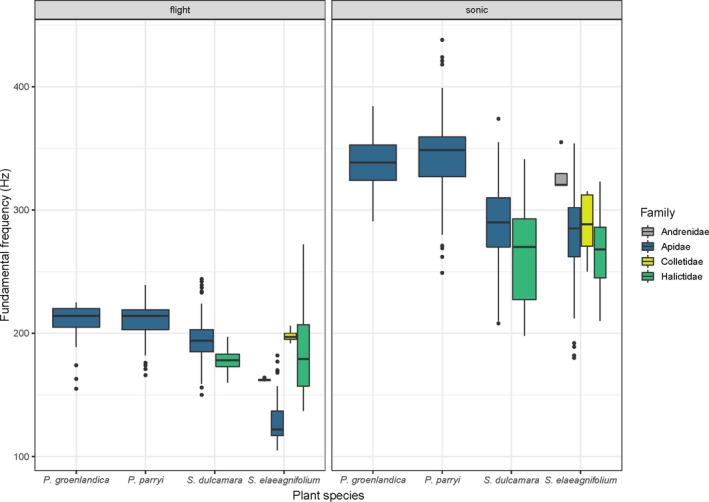
Fundamental frequency of flight (flight) and floral sonication (sonic) vibrations in bees visiting four buzz‐pollinated plant species in North America: *Pedicularis groenlandica*, *P. parryi* (Orobanchaceae), *Solanum dulcamara*, and *S. elaeagnifolium* (Solanaceae). We show data for bees within the Andrenidae and Colletidae, but did not include these families in our statistical analyses (see text). Presentation order of bee families is the same in both panels

**Figure 4:**
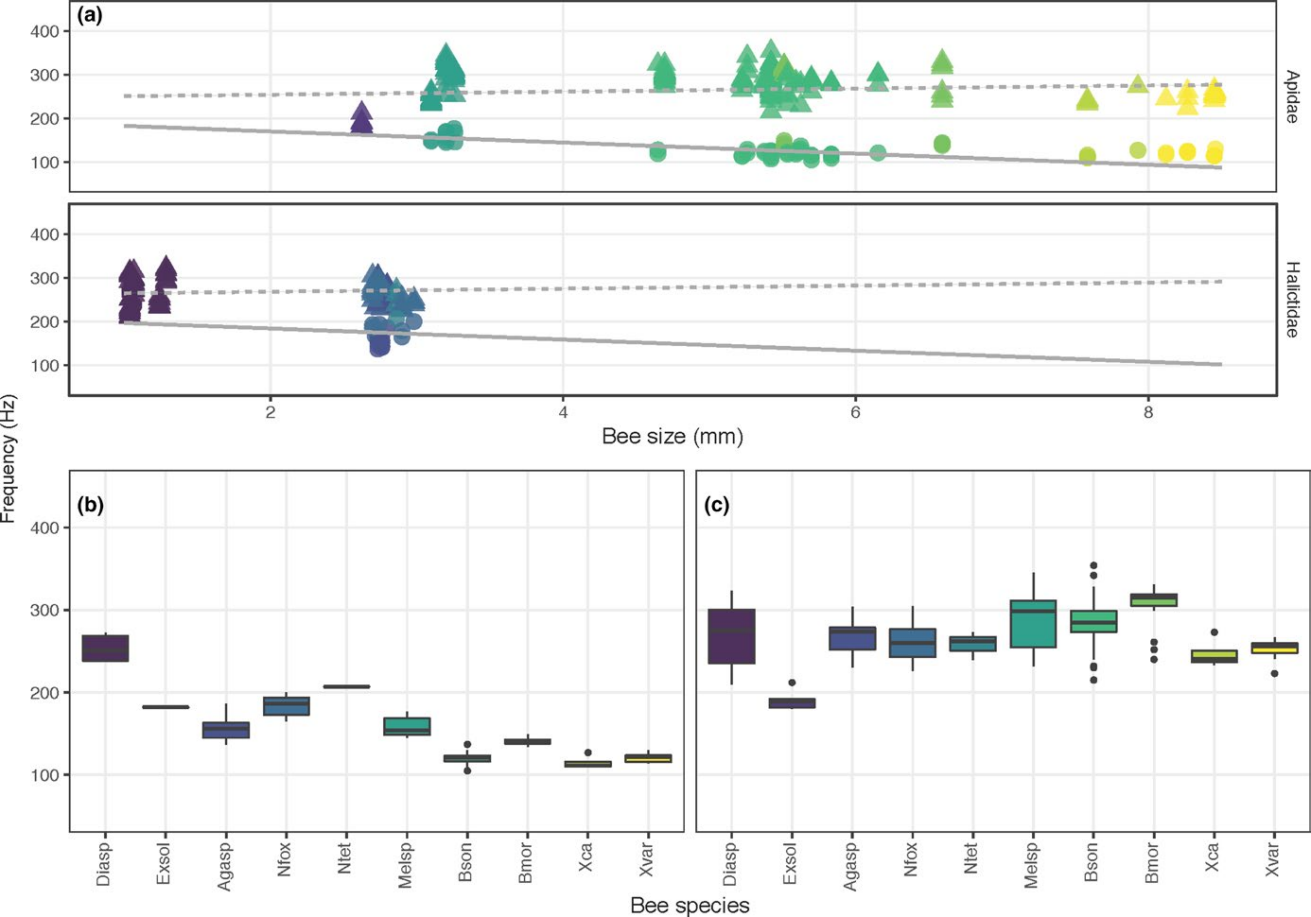
(a) Relationship between fundamental frequency (cycles per second, Hz) and bee size (ITD, mm) in bees from Apidae and Halictidae visiting *Solanum elaeagnifolium* (Solanaceae) in three localities in Arizona and New Mexico. Each data point corresponds to a single vibration. Circles and solid line: flight; triangles and dashed line: floral sonication. The regression lines show predicted values from the statistical model. (b, c) Boxplots of the same data grouped by bee species. Bee species and vibration type (b: flight; c: floral) are arranged from smallest (*Dialictus* sp., Diasp) to largest (*Xylocopa varipuncta*, Xvar)

**Figure 5:**
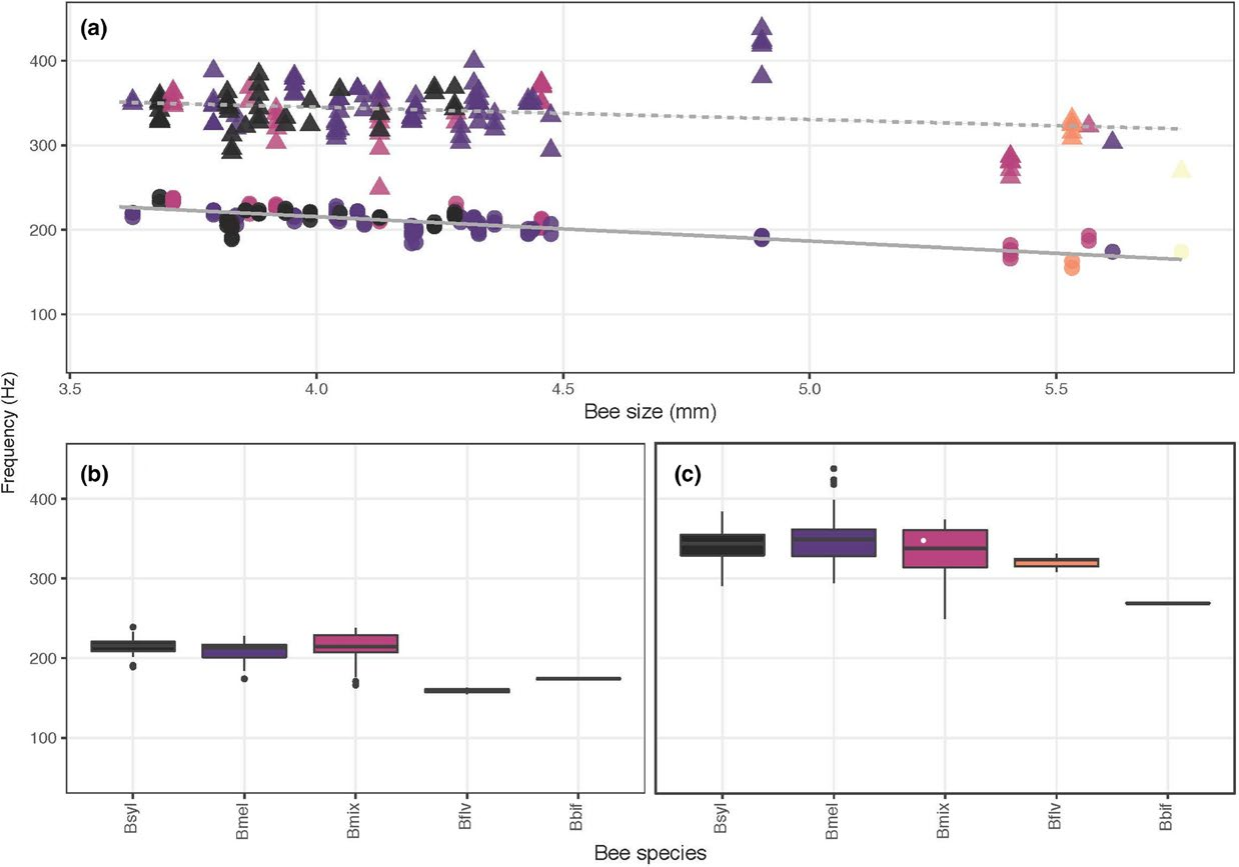
(a) Relationship between fundamental frequency (cycles per second, Hz) and bee size (ITD, mm) in bumblebees (*Bombus* spp.) visiting two species of *Pedicularis* (*P. groenlandica *and *P. parryi*, Orobanchaceae) in a single locality in Colorado. Each data point corresponds to a single vibration. Circles and solid line: flight; triangles and dashed line: floral sonication. The regression lines show predicted values from the statistical model. (b, c) Boxplots of the same data grouped by bee species and vibration type (b: flight; c: floral) are arranged from smallest (*B. sylvicola*, Bsyl) to largest (*B. bifarius*, Bbif)

**Figure 6:**
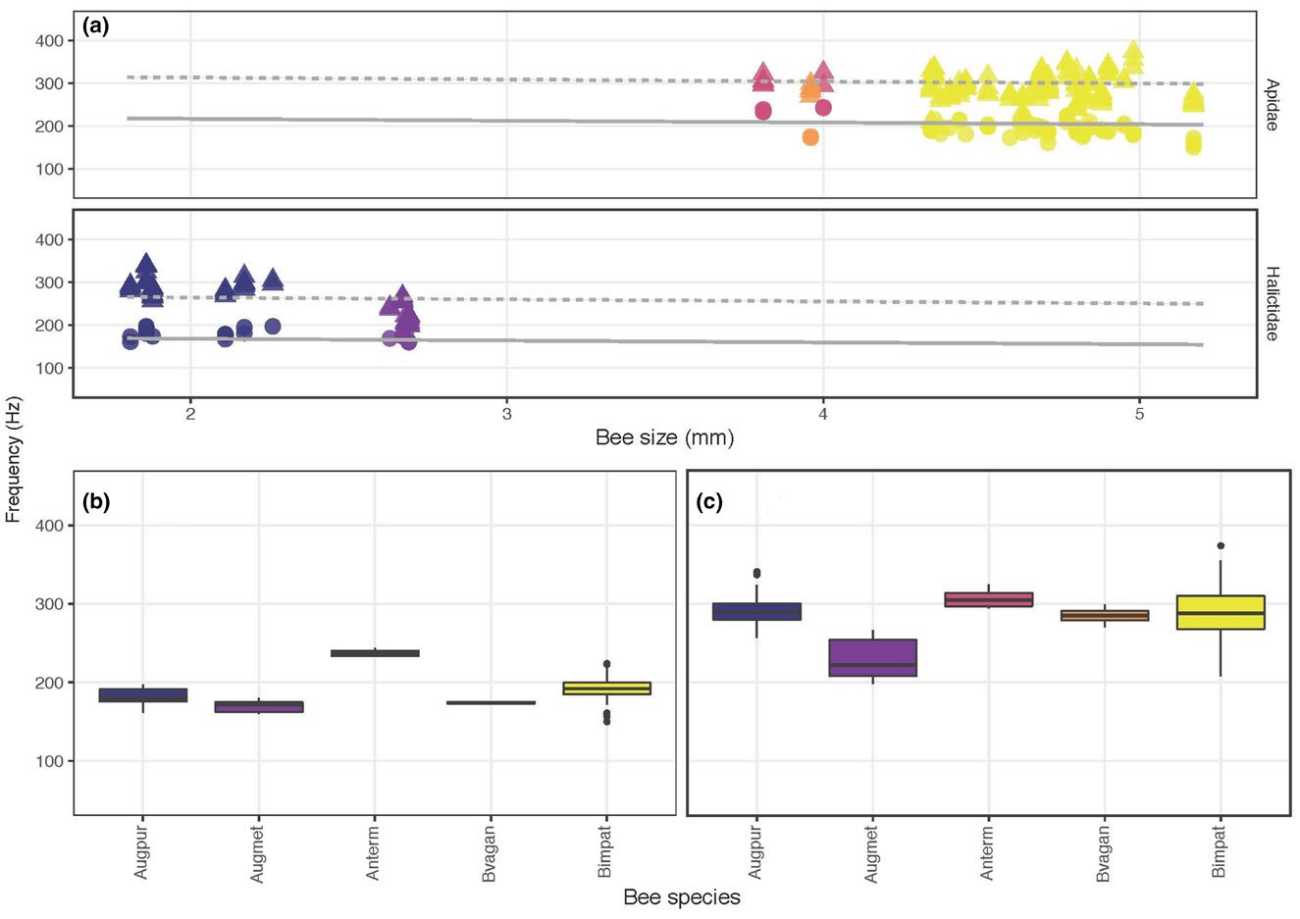
(a) Relationship between fundamental frequency (cycles per second, Hz) and bee size (ITD, mm) in bees from Apidae and Halictidae visiting *Solanum dulcamara* (Solanaceae) in southern Ontario, Canada. Each data point corresponds to a single vibration. Circles and solid line: flight; triangles and dashed line: floral sonication. The regression lines show predicted values from the statistical model. (b, c) Boxplots of the same data grouped by bee species and vibration type (b: flight; c: floral) are arranged from smallest (*Auguchlora pura*, Augpur) to largest (*Bombus impatiens*, Bimpat)

The fundamental vibration frequencies of floral vibrations were higher than those of flight regardless of location or plant host (Figure 3). On average, the fundamental frequency of both floral and flight vibrations was higher in bees visiting the two *Pedicularis* species in Colorado than in those visiting *S. elaeagnifolium* in Arizona/New Mexico and *S. dulcamara* in southern Ontario, Canada (Figure 3). For *S. elaeagnifolium*, floral sonications had significantly higher frequencies than flight vibrations, and bee size (ITD) was negatively associated with fundamental frequency (Table 2A). However, we detected a significant interaction between bee size and the type of vibration (flight or floral). Interestingly, the slope of the relationship between frequency and size was steeper (more negative) for flight vibrations and became shallower, and slightly positive, for floral sonication vibrations (Table 2A, Figure 4). Finally, although the flight frequency in Halictidae was, on average, higher than in Apidae (189 vs. 128 Hz, respectively), the frequency of floral sonication vibrations was lower in Halictidae (267 vs. 282 Hz, respectively; Table 2A, Figure 4). For *P. groenlandica* and *P. parryi*, sonication frequencies were significantly higher than flight frequencies (340 vs. 209 Hz respectively; Table 2B). Bee size was negatively correlated with the fundamental frequency of bees’ vibrations during both flight and floral sonication, but as in *S. elaeagnifolium*, we found a significant interaction between size and buzz type. The negative relationship between frequency and bee size was shallower for floral sonication vibrations than for flight vibrations (Figure 5). Finally, for S*. dulcamara*, we also found a significant difference in the fundamental frequency of floral sonication vibrations and flight, with floral vibrations (Halictidae = 289 Hz, Apidae = 265 Hz) being higher than flight vibrations (Halictidae = 196 Hz, Apidae = 178 Hz) (Table 2C). However, although the relationship with size was slightly negative, it was not statistically significant with either flight or floral sonication frequency (Table 2C, Figure 6).

**Table 2:** Parameter estimates and analysis of variance of the fundamental frequency of floral sonication and flight vibrations of bees in the families Apidae and Halictidae visiting flowers of *Solanum spp*. or *Pedicularis* spp. (A) *Solanum elaeagnifolium*. (B) *Pedicularis groenlandica *and *P. parryi*. Note that for *Pedicularis *we only observed bumblebees (*Bombus* spp., Apidae). (C) *Solanum dulcamara*

Parameter	Estimate	*SE*	*p*‐Value
(A)			
Intercept (Apidae, flight)	195.566	26.092	
Buzz type (floral sonication)	51.794	7.069	<0.0001
Bee size (ITD)	−12.713	4.570	0.340
Family (Halictidae)	14.030	21.828	0.555
Buzz type*Bee size	16.206	1.408	<0.0001
(B)			
Intercept (flight)	330.971	19.552	
Buzz type (floral sonication)	72.852	18.105	<0.0001
Bee size (ITD)	−28.861	4.600	<0.0001
Buzz type*Bee size	14.182	4.272	0.001
(C)			
Intercept (Apidae, flight)	225.380	66.3138	
Buzz type (floral sonication)	97.275	8.480	<0.0001
Bee size (ITD)	−4.281	14.596	0.761
Family (Halictidae)	−48.738	40.764	0.282
Buzz type*Bee size	−0.353	2.039	0.862

Note

Statistical significance was assessed for each explanatory variable using a Type III Analysis of Variance with Sattertwhaite's method as implemented in *lmerTest*.

### 3.2 Floral vibration duration and bee size

We found a small, but significant positive association between bee size and the duration of individual floral sonication vibrations in bees visiting *S. elaeagnifolium* (regression coefficient = 0.056, *p* = 0.043). Size was not significantly associated with duration for bees visiting *Pedicularis *spp. (regression coefficient = 0.132, *p* = 0.239) or *S. dulcamara* (regression coefficient = 0.204, *p* = 0.366). The duration of floral vibrations was not significantly different between bees in the families Apidae and Halictidae foraging on *S. elaeagnifolium* (coefficient = −0.032, *p* = 0.754), or on *S. dulcamara* (coefficient = 0.085, *p* = 0.877).

### 3.3 Buzz ratio and bee size

**Figure 7:**
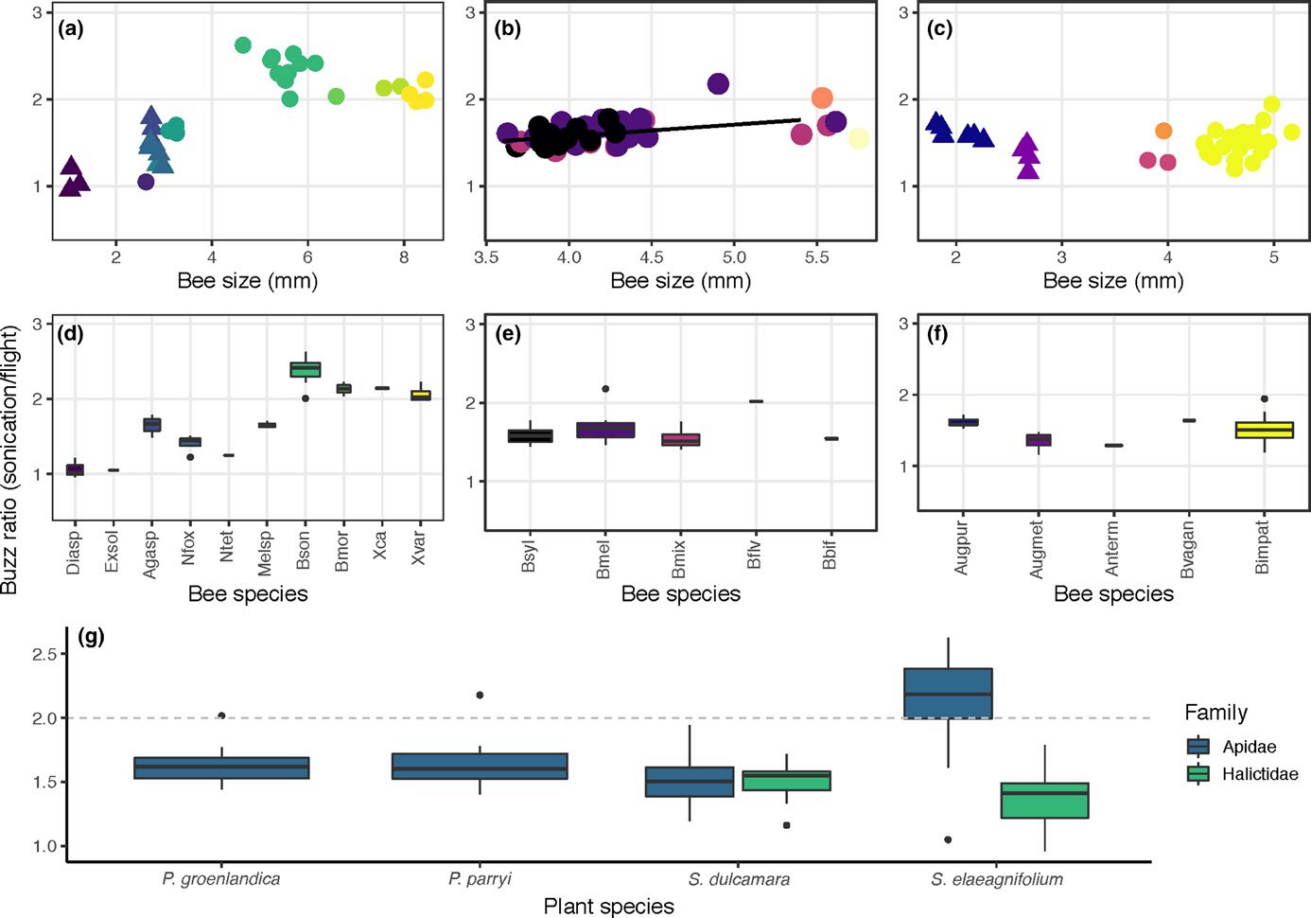
Buzz ratio, defined as the fundamental frequency of floral sonication vibration divided by the fundamental frequency of flight vibration, for bees in the families Apidae and Halictidae, visiting flowers of *Solanum elaeagnifolium, Pedicularis groenlandica*, *P. parryi*, or *S. dulcamara*. Each data point corresponds to the buzz ratio for a single bee. Buzz ratio for bees visiting *S. elaeagnifolium *is shown in panels a and d, for *P. groenlandica *and *P. parryi *in panels b and e, and for *S. dulcamara *in panels c and f. Panels a–c show the relationship between buzz ratio and bee size (ITD, mm). Panels d–f show boxplots of the same data grouped by bee species. Bee species are arranged from smallest to largest. Panel g shows the buzz ratio across all bees in Apidae and Halictidae for each of the plant species studied. The dashed line corresponds to a buzz ratio of 2.0, that is, floral vibration frequency is double the flight vibration frequency

For bees in Arizona/New Mexico, buzz ratios ranged from 0.95 to 2.62 (mean = 1.85, *SD* = 0.48; Figure 7a,d). We observed a strong positive association between buzz ratio and size, but the association was not statistically significant when we accounted for bee genus and species in the linear mixed‐effect model (coefficient for ITD = −0.114, *p* = 0.107). Buzz ratio was, on average, lower for bees in the family Halictidae than in Apidae, but this difference was not statistically significant when accounting for genus and species as random effects (coefficient for Halictidae = −0.731, *p* = 0.263, Figure 7g). For bees in Colorado, buzz ratios ranged from 1.40 to 2.18 (mean = 1.63, *SD* = 0.15; Figure 7b,e). We found a positive association between buzz ratio and size, which was significant in the linear mixed‐effect model (coefficient = 0.134, *p* = 0.002). For bees in southern Ontario, buzz ratios ranged from 1.61 to 1.94 (mean = 1.49, *SD* = 0.16; Figure 7c,f). There was no significant association between buzz ratio and size in the linear mixed‐effect model (coefficient = 0.137, *p* = 0.227). We also did not detect any significant difference in buzz ratio between bees in the families Apidae and Halictidae when accounting for genus and species as random effects (coefficient = 0.300, *p* = 0.335, Figure 7g).

## 4 DISCUSSION

Our study reveals consistent patterns regarding the influence of body size on the frequency of floral sonication vibrations across the different environments. We expected body size to be strongly negatively correlated with flight frequency as is commonly reported in the literature for flying insects, and this was confirmed in bees from Colorado and Arizona/New Mexico, but not in southern Ontario (although the trend here was still negative). However, across all sites, the relationship between body size and floral vibration frequency was much weaker than between size and flight frequency, revealing that floral vibration frequencies are not strongly associated with body size. Furthermore, smaller bees did not significantly increase the fundamental frequency of their floral vibrations compared to larger bees as proposed by Corbet and Huang (2014). Although there were two exceptions of smaller bees having buzz ratios comparable to larger bees (e.g., *Agapostemon femoratus* in Arizona/New Mexico— Figure 7d, *Augochlora pura* in Ontario— Figure 7f), for the most part, buzz ratios increased with body size such that only the largest bees (e.g., apids in the genera *Bombus*, *Melissodes*, and *Xylocopa*) were capable of generating floral vibrations using frequencies that approached or exceeded twice that of their flight vibration frequency. Interestingly, although the association between buzz ratio and size for bees in Arizona/New Mexico was positive and strong it was not statistically significant when we accounted for species, genus, and family in the model. This was likely due to overall size differences between Apidae and Halictidae explaining most of the association between buzz ratio and size (compare the distribution of data points for Apidae (circles) and Halictidae (triangles) in Figure 7a). Nevertheless, our results show that only the larger individuals of species within the Apidae are capable of raising their floral vibration frequency substantially above their flight frequency.

In the three other studies that calculated buzz ratio values, larger bees also had higher ratios than smaller bees (Burkart et al., 2011; Corbet & Huang, 2014; King & Buchmann, 2003). Accordingly, smaller bees appear unable to raise sonication vibration frequencies substantially above their flight frequencies. Why might this be so? One possible explanation is that generating sonication frequencies that approach or exceed double that of flight frequencies entails greater physiological costs for smaller bees. To our knowledge, the energetics of floral sonication behavior has not been evaluated, but studies examining the energetics of insect flight have shown that smaller individuals have higher flight frequencies and expend greater energy when flying (or hovering) than larger bodied insects (Casey et al., 1985; Ellington, 1985; Tercel, Veronesi, & Pope, 2018). Thus, for a smaller bee, trying to raise sonication frequency significantly above an already high flight frequency may be energetically more difficult than it is for a larger bee that is starting off at a much lower flight frequency. In fact, regardless of body size, the maximum value of sonication fundamental frequencies reported for bees is about 400 Hz (Arroyo‐Correa et al., 2019; Burkart et al., 2011; Corbet, Chapman, & Saville, 1988; De Luca & Vallejo‐Marín, 2013; King, 1993; Macior, 1968; Rosi‐Denadai et al., 2018; Switzer & Combes, 2017), including the new data presented here. This value may thus represent an upper physiological limit for producing floral sonication vibrations regardless of body size. Larger bees can easily approach this when doubling their low flight frequencies, but smaller bees, by virtue of having higher flight frequencies, are closer to this limit and thus cannot exceed it. One consequence of this is that larger bees may have an advantage when foraging on buzz‐pollinated flowers because even at the maximum value of 400 Hz, larger bees are also capable of achieving greater thoracic displacements (Corbet & Huang, 2014; King & Buchmann, 2003). Accordingly, they can generate floral vibrations with greater amplitudes than smaller bees, and thus remove comparatively more pollen for the same foraging effort. Although smaller bees might compensate by adjusting other aspects of their foraging behavior (e.g., increasing the duration of floral vibrations) to increase pollen collection (Buchmann & Cane, 1989; Rosi‐Denadai et al., 2018; Russell, Buchmann, & Papaj, 2017; Switzer & Combes, 2017), all else being equal, the ability of larger bees to maximize frequency and displacement gives them a physical advantage when buzz‐pollinating that smaller bees cannot match.

The flat relationship between body size and floral vibration frequency observed in all ecoregions is consistent with the hypothesis that bees are converging on a common frequency range when foraging on the same buzz‐pollinated plant host (Switzer & Combes, 2017). However, our results do not allow us to conclusively test whether this is indeed occurring, or if the pattern we observed is due to greater variance in floral vibration frequency values, which would tend to flatten its relationship with body size. Because our study sampled bees as they were foraging naturally, there were likely many differences between individuals in a range of factors (e.g., physical condition, age, and experience). Furthermore, bees refine buzz‐pollination behavior with experience (Buchmann & Cane, 1989; Morgan, Whitehorn, Lye, & Vallejo‐Marín, 2016), thus we had no way of knowing whether some bees were more experienced foragers than others when visiting the same buzz‐pollinated plant, and how this might have influenced the frequencies they used. Accordingly, to properly evaluate the frequency convergence hypothesis, a laboratory experiment is needed that uses flower‐naïve bees whose floral vibration behavior can be measured as it first develops, and then is monitored over time as individuals gain foraging experience.

Although we found some differences between ecoregions in both flight and floral vibration frequency ranges that bees used, our study does not allow us to identify causal factors explaining why these differences existed between environments. Because neither plants nor bees overlapped in the different areas, it was impossible to compare flight or floral vibration properties of the same bee species foraging in different environments. Nevertheless, habitat‐specific effects might still influence the frequency ranges of floral vibrations. For example, at our alpine site, both flight and floral vibration frequencies were significantly higher than in our desert and grassland sites, even though the body size ranges of bees from Colorado overlapped with that for bees from both Arizona/New Mexico and southern Ontario. This may not be unexpected, however, as studies show that at colder temperatures flying insects tend to have higher flight frequencies (Esch, 1985; Harrison & Fewell, 2002; Unwin & Corbet, 1984), which in Colorado may represent a high elevation cold climate adaptation (Addo‐Bediako, Chown, & Gaston, 2002). In our desert sites, both flight and floral vibration frequencies were lower than in the other two ecogeographic locations, and this could be a result of physiological adaptations in hotter climates to prevent overheating in flying insects (Harrison & Fewell, 2002; Harrison, Fewell, Roberts, & Hall, 1996; Roberts & Harrison, 1998).

Our findings have important implications that expand our understanding of the role body size plays in mediating plant – pollinator behavioral interactions. Although different assemblages of bees and plants existed in the three ecogeographic regions, the patterns we observed were similar and also congruent with those from a tropical locale in Brazil (Burkart et al., 2011). Accordingly, we have identified some robust relationships within the buzz‐pollination syndrome. First, the frequencies used to generate floral vibrations are not tightly linked with body size, which may allow an individual bee to use different sonication frequencies when visiting different kinds of buzz‐pollinated flowers (Arroyo‐Correa et al., 2019; Switzer & Combes, 2017), and thus take full advantage of the suite of plant species that may be available in a given habitat. This may promote a more generalized plant‐pollinator community assemblage within this syndrome in a given habitat (De Luca et al., 2013; Larson & Barrett, 1999; Rosi‐Denadai et al., 2018). Second, the relationship between body size and buzz ratio reveals that larger bees may have an advantage when visiting a buzz‐pollinated flower due to their ability to maximize the amplitude of their floral vibrations, and thus extract higher levels of pollen for their foraging effort. Consequently, we have identified three key areas for future research: (a) examining the extent to which an individual bee adjusts its floral vibration frequencies when visiting different kinds of buzz‐pollinated flowers, (b) determining the environmental factors that cause variability in properties of floral vibrations between different habitats (e.g., alpine vs. desert), and (c) evaluating how size‐dependent variation in properties of floral vibrations correlates with the amount of pollen individual bees collect from flowers, and the resulting fitness consequences for both plant and pollinator.

## CONFLICT OF INTEREST

None declared.

## AUTHOR CONTRIBUTIONS

PAD, ACM and MVM conceived the ideas and designed methodology; PAD, CG and SB collected the data; PAD and MVM analyzed the data; PAD and MVM led the writing of the manuscript. All authors contributed critically to the drafts and gave final approval for publication.

## Data Availability

Data associated with this paper is available in the Dryad Digital Repository: https://doi.org/10.5061/dryad.6g8qr82.

## References

[ece35092-bib-0001] Addo‐Bediako, A. , Chown, S. L. , & Gaston, K. J. (2002). Metabolic cold adaptation in insects: A large‐scale perspective. Functional Ecology, 16, 332–338. 10.1046/j.1365-2435.2002.00634.x

[ece35092-bib-0002] Anderson, B. , Pauw, A. , Cole, W. W. , & Barrett, S. C. H. (2016). Pollination, mating and reproductive fitness in a plant population with bimodal floral‐tube length. Journal of Evolutionary Biology, 29, 1631–1642. 10.1111/jeb.12899 27206242

[ece35092-bib-0003] Anderson, B. , Terblanche, J. S. , & Ellis, A. G. (2010). Predictable patterns of trait mismatches between interacting plants and insects. BMC Evolutionary Biology, 10, 204 10.1186/1471-2148-10-204 20604973 PMC2927919

[ece35092-bib-0004] Arceo‐Gómez, G. , Martinez, M. L. , Parra‐Tabla, V. , & Garcıa‐Franco, J. G. (2011). Anther and stigma morphology in mirror‐image flowers of *Chamaecrista chamaecristoides* (Fabaceae): Implications for buzz pollination. Plant Biology, 13(Suppl 1), 19–24. 10.1111/j.1438-8677.2010.00324.x 21134083

[ece35092-bib-0005] Arroyo‐Correa, B. , Beattie, C. , & Vallejo‐Marín, M. (2019). Bee and floral traits affect the characteristics of the vibrations experienced by flowers during buzz‐pollination. Journal of Experimental Biology, 10.1242/jeb.198176 30760551

[ece35092-bib-0006] Bates, D. , Maechler, M. , & Bolker, B. (2014). lme4: Linear mixed‐effects models using Eigen and S4. R package version 1.1‐7. Retrieved from http://CRAN.R-project.org/package=lme4

[ece35092-bib-0007] Buchmann, S. L. (1983). Buzz pollination in angiosperms In Jones C. E., & Little R. J. (Eds.), Handbook of experimental pollination biology (pp. 73–113). New York, NY: van Nostrand Reinhold Company Inc.

[ece35092-bib-0008] Buchmann, S. L. , & Cane, J. H. (1989). Bees assess pollen returns while sonicating *Solanum* flowers. Oecologia, 81, 289–294. 10.1007/BF00377073 28311178

[ece35092-bib-0009] Buchmann, S. L. , & Hurley, J. P. (1978). Biophysical model for buzz pollination in Angiosperms. Journal of Theoretical Biology, 72, 639–657. 672247 10.1016/0022-5193(78)90277-1

[ece35092-bib-0010] Burkart, A. , Lunau, K. , & Schlindwein, C. (2011). Comparative bioacoustical studies on flight and buzzing of neotropical bees. Journal of Pollination Ecology, 6, 118–124.

[ece35092-bib-0011] Byrne, D. , Buchman, S. L. , & Spangler, H. G. (1988). Relationship between wing loading, wingbeat frequency and body mass in homopterous insects. Journal of Experimental Biology, 135, 9–23.

[ece35092-bib-0012] Cane, J. H. (1987). Estimation of bee size using intertegular span (Apoidea). Journal of the Kansas Entomological Society, 60, 145–147.

[ece35092-bib-0013] Cardinal, S. , Buchmann, S. L. , & Russell, A. L. (2018). The evolution of floral sonication, a pollen foraging behavior used by bees (Anthophila). Evolution, 72, 590–600. 10.1111/evo.13446 29392714 PMC5873439

[ece35092-bib-0014] Casey, T. M. , May, M. L. , & Morgan, K. R. (1985). Flight energetics of euglossine bees in relation to morphology and wing stroke frequency. Journal of Experimental Biology, 116, 271–289.

[ece35092-bib-0015] Chown, S. L. , & Gaston, K. J. (2010). Body size variation in insects: A macroecological perspective. Biological Reviews, 85, 139–169. 10.1111/j.1469-185X.2009.00097.x 20015316

[ece35092-bib-0016] Corbet, S. A. , Chapman, H. , & Saville, N. (1988). Vibratory pollen collection and flower form: Bumble‐bees on *Actinidia*, *Symphytum*, *Borago* and *Polygonatum* . Functional Ecology, 2, 147–155. 10.2307/2389689

[ece35092-bib-0017] Corbet, S. A. , & Huang, S. Q. (2014). Buzz pollination in eight bumblebee‐pollinated *Pedicularis* species: Does it involve vibration‐induced triboelectric charging of pollen grains? Annals of Botany, 114, 1665–1674. 25274550 10.1093/aob/mcu195 PMC4649695

[ece35092-bib-0018] De Luca, P. A. , Bussière, L. F. , Souto‐Vilaros, D. , Goulson, D. , Mason, A. C. , & Vallejo‐Marín, M. (2013). Variability in bumblebee pollination buzzes affects the quantity of pollen released from flowers. Oecologia, 172, 805–816. 10.1007/s00442-012-2535-1 23188056

[ece35092-bib-0019] De Luca, P. A. , Cox, D. A. , & Vallejo‐Marín, M. (2014). Comparison of pollination and defensive buzzes in bumblebees indicates species‐specific and context‐dependent vibrations. Naturwissenschaften, 101, 331–338. 10.1007/s00114-014-1161-7 24563100

[ece35092-bib-0020] De Luca, P. A. , Giebink, N. , Mason, A. C. , Papaj, D. R. , & Buchmann, S. L. (2018). How well do acoustic recordings characterize properties of bee (Anthophila) floral sonication vibrations? Bioacoustics, 10.1080/09524622.2018.1511474

[ece35092-bib-0021] De Luca, P. A. , & Vallejo‐Marín, M. (2013). What's the buzz about? The ecology and evolutionary significance of buzz‐pollination. Current Opinion in Plant Biology, 16, 429–435. 10.1016/j.pbi.2013.05.002 23751734

[ece35092-bib-0022] Dellinger, A. S. , Chartier, M. , Fernández‐Fernández, D. , Penneys, D. S. , Alvear, M. , Almeda, F. , … Schönenberger, J. (2018). Beyond buzz‐pollination – departures from an adaptive plateau lead to new pollination syndromes. New Phytologist, 221, 1136–1149. 30368819 10.1111/nph.15468 PMC6492237

[ece35092-bib-0023] Ellington, C. P. (1985). Power and efficiency of insect flight muscle. Journal of Experimental Biology, 115, 293–304. 4031771 10.1242/jeb.115.1.293

[ece35092-bib-0024] Esch, H. (1985). The effects of temperature on flight muscle potentials in honeybees and cuculiinid winter moths. Journal of Experimental Biology, 135, 109–117.

[ece35092-bib-0025] Gilmour, K. M. , & Ellington, C. P. (1993). *In vivo* muscle length changes in bumblebees and the *in vitro* effects on work and power. Journal of Experimental Biology, 183, 101–113.

[ece35092-bib-0026] Greenleaf, S. S. , Williams, N. N. , Winfree, R. , & Kremen, C. (2007). Bee foraging ranges and their relationship to body size. Oecologia, 153, 589–596. 10.1007/s00442-007-0752-9 17483965

[ece35092-bib-0027] Harder, L. D. (1985). Morphology as a predictor of flower choice by bumble bees. Ecology, 66, 198–210. 10.2307/1941320

[ece35092-bib-0028] Harder, L. D. , & Barclay, M. R. (1994). The functional significance of poricial anthers and buzz pollination: Controlled pollen removal from *Dodecatheon* . Functional Ecology, 8, 509–517.

[ece35092-bib-0029] Harrison, J. F. , & Fewell, J. H. (2002). Environmental and genetic influences on flight metabolic rate in the honey bee, *Apis mellifera* . Comparative Biochemistry and Physiology, Part A: Molecular & Integrative Physiology, 133, 323–333. 10.1016/S1095-6433(02)00163-0 12208303

[ece35092-bib-0030] Harrison, J. F. , Fewell, J. H. , Roberts, S. P. , & Hall, H. G. (1996). Achievement of thermal stability by varying metabolic heat production in flying honeybees. Science, 274, 88–90. 10.1126/science.274.5284.88 8810252

[ece35092-bib-0031] Josephson, R. K. , Malamud, J. G. , & Stokes, D. R. (2000). Asynchronous muscle: A primer. Journal of Experimental Biology, 203, 2713–2722. 10952872 10.1242/jeb.203.18.2713

[ece35092-bib-0032] King, M. J. (1993). Buzz foraging mechanism in bumble bees. Journal of Apicultural Research, 32, 41–49.

[ece35092-bib-0033] King, M. J. , & Buchmann, S. L. (1995). Bumble bee‐initiated vibration release mechanism of *Rhododendron* pollen. American Journal of Botany, 82, 1407–1411.

[ece35092-bib-0034] King, M. J. , & Buchmann, S. L. (1996). Sonication dispensing of pollen from *Solanum laciniatum* flowers. Functional Ecology, 10, 449–456. 10.2307/2389937

[ece35092-bib-0035] King, M. J. , & Buchmann, S. L. (2003). Floral sonication by bees: Mesosomal vibration by *Bombus* and *Xylocopa*, but not *Apis* (Hymenoptera: Apidae), ejects pollen from poricidal anthers. Journal of the Kansas Entomological Society, 76, 295–305.

[ece35092-bib-0036] King, M. J. , Buchmann, S. L. , & Spangler, H. G. (1996). Activity of asynchronous flight muscle from two bee families during sonication (buzzing). Journal of Experimental Biology, 199, 2317–2321. 8896367 10.1242/jeb.199.10.2317

[ece35092-bib-0037] King, M. J. , & Lengoc, L. (1993). Vibratory pollen collection dynamics. Transactions of the American Society of Agricultural Engineers, 36, 135–140. 10.13031/2013.28324

[ece35092-bib-0038] Kuznetsova, A. , Brockhoff, P. B. , & Christensen, R. H. B. (2016). lmerTest: Tests in linear mixed effect models. R package version 2.0‐30.

[ece35092-bib-0039] Larson, B. M. , & Barrett, S. C. H. (1999). The pollination ecology of buzz‐pollinated *Rhexia virginica *(Melastomataceae). American Journal of Botany, 86, 502–511. 10205070

[ece35092-bib-0040] Lüdeke, D. (2017). sjPlot: Data visualization for statistics in social science. R Package Version 2.3. 3.

[ece35092-bib-0041] Macior, L. W. (1968). Pollen adaptation in *Pedicularis groenlandica* . American Journal of Botany, 55, 927–932.

[ece35092-bib-0042] Miller‐Struttmann, N. E. , Geib, J. C. , Franklin, J. D. , Kevan, P. G. , Holdo, R. M. , Ebert‐May, D. , … Galen, C. (2015). Functional mismatch in a bumble bee pollination mutualism under climate change. Science, 349, 1541–1544. 10.1126/science.aab0868 26404836

[ece35092-bib-0043] Molloy, J. E. , Kyrtatas, V. , Sparrow, J. C. , & White, D. C. S. (1987). Kinetics of flight muscles from insects with different wingbeat frequencies. Nature, 328, 449–451. 10.1038/328449a0

[ece35092-bib-0044] Morgan, T. , Whitehorn, P. R. , Lye, G. C. , & Vallejo‐Marín, M. (2016). Floral sonication is an innate behaviour in bumblebees that can be fine‐tuned with experience in manipulating flowers. Journal of Insect Behavior, 29, 233–241. 10.1007/s10905-016-9553-5 27194824 PMC4841848

[ece35092-bib-0045] Nunes‐Silva, P. , Hrncir, M. , Shipp, L. , Kevan, P. , & Imperatriz‐Fonseca, V. L. (2013). The behaviour of *Bombus impatiens* (Apidae, Bombini) on tomato (*Lycopersicon esculentum* Mill., Solanaceae) flowers: Pollination and reward perception. Journal of Pollination Ecology, 11, 33–40.

[ece35092-bib-0046] Peat, J. , Tucker, J. , & Goulson, D. (2005). Does intraspecific size variation in bumblebees allow colonies to efficiently exploit different flowers? Ecological Entomology, 30, 176–181.

[ece35092-bib-0047] Pringle, J. W. S. (1949). The excitation and contraction of the flight muscles of insects. Journal of Physiology, 108, 226–232. 10.1113/jphysiol.1949.sp004326 16991854 PMC1392366

[ece35092-bib-0048] R Core Team (2018). R: A language and environment for statistical computing. Vienna, Austria: R Foundation for Statistical Computing Retrieved from http://www.R-project.org/

[ece35092-bib-0049] Renauld, M. , Hutchinson, A. , Loeb, G. , Poveda, K. , & Connelly, H. (2016). Landscape simplification constrains adult size in a native ground‐nesting bee. PLoS ONE, 11(3), e0150946 10.1371/journal.pone.0150946 26943127 PMC4778946

[ece35092-bib-0050] Roberts, S. P. , & Harrison, J. F. (1998). Mechanisms of thermoregulation in flying bees. American Zoologist, 38, 492–502. 10.1093/icb/38.3.492

[ece35092-bib-0051] Rosi‐Denadai, C. A. , Araújo, P. C. S. , de Oliveira Campos, L. A. , Cosme, L. Jr , & Guedes, R. N. C. (2018). Buzz‐pollination in Neotropical bees: Genus‐dependent frequencies and lack of optimal frequency for pollen release. Insect Science, 10.1111/1744-7917.12602 29740981

[ece35092-bib-0052] Russell, A. L. , Buchmann, S. L. , & Papaj, D. R. (2017). How a generalist bee achieves high efficiency of pollen collection on diverse floral resources. Behavioral Ecology, 28, 991–1003. 10.1093/beheco/arx058

[ece35092-bib-0053] Solis‐Montero, L. , & Vallejo‐Marin, M. (2017). Does the morphological fit between flowers and pollinators affect pollen deposition? An experimental test in a buzz‐pollinated species with anther dimorphism. Ecology and Evolution, 7, 2706–2715. 10.1002/ece3.2897 28428861 PMC5395427

[ece35092-bib-0054] Switzer, C. , & Combes, S. (2017). Bumblebee sonication behavior changes with plant species and environmental conditions. Apidologie, 48, 223–233. 10.1007/s13592-016-0467-1

[ece35092-bib-0055] Tercel, M. P. , Veronesi, F. , & Pope, T. W. (2018). Phylogenetic clustering of wingbeat frequency and flight‐associated morphometrics across insect orders. Physiological Entomology, 43, 149–157. 10.1111/phen.12240

[ece35092-bib-0056] Unwin, D. M. , & Corbet, S. A. (1984). Wingbeat frequency, temperature and body size in bees and flies. Physiological Entomology, 9, 115–121. 10.1111/j.1365-3032.1984.tb00687.x

[ece35092-bib-0057] Vallejo‐Marín, M. (2019). Buzz pollination: Studying bee vibrations on flowers. New Phytologist, 10.1111/nph.15666 30585638

[ece35092-bib-0058] White, E. P. , Ernest, S. M. , Kerkhoff, A. J. , & Enquist, B. J. (2007). Relationships between body size and abundance in ecology. Trends in Ecology & Evolution, 22, 323–330. 10.1016/j.tree.2007.03.007 17399851

[ece35092-bib-0059] Woodward, G. , Ebenman, B. , Emmerson, M. , Montoya, J. M. , Olesen, J. M. , Valido, A. , & Warren, P. H. (2005). Body size in ecological networkds. Trends in Ecology & Evolution, 20, 402–409. 16701403 10.1016/j.tree.2005.04.005

[ece35092-bib-0060] Zurbuchen, A. , Landert, L. , Klaiber, J. , Müller, A. , Hein, S. , & Dorn, S. (2010). Maximum foraging ranges in solitary bees: Only few individuals have the capability to cover long foraging distances. Biological Conservation, 143, 669–676. 10.1016/j.biocon.2009.12.003

